# On the Corrective Influence of Bromide of Potassium on Opium

**Published:** 1871-04

**Authors:** J. M. Da Costa

**Affiliations:** Physician to the Pennsylvania Hospital, Etc.


### On the Corrective Influence of Bromide of Potassium on Opium.

J. M. DA COSTA, M. D., PHYSICIAN TO THE PENNSYLVANIA HOSPITAL, ETC.

In an article in this Journal for April, 1870, (p. 365), I called attention to the influence bromide of potassium exerts on the unpleasant effects produced by opium. I propose here to give some cases which led me to form this opinion, and to examine more particularly into the combined action of these valuable agents.

The first case in which I fairly studied the subject was under my charge about two years ago. A lady affected with a most painful enteric malady, and of very susceptible nervous system, was often attacked with seizures of abdominal pain of most serious character. Yet she generally bore them until they exhausted themselves without taking any remedy, or with such slight help as remedies, excepting opium, afforded, rather than subject herself to the distress this medicine caused. It was not fancy on her part; for when at times, on account of the excruciating character of the pain, she was obliged to resort to opium—usually black drop, which, of all preparations, produced the least disturbance—I have stood by her bedside and witnessed the effects of the anodyne. There was relief, certainly, of the abdominal distress, but also itching or tingling sensations all over the body, amounting to positive pain; then numbness more or less extended, usually accompanied by a sense of sinking, and a faintness most severe and constant, and uninfluenced, or with difficulty relieved by stimulants. Complete unconsciousness did not occur, or only existed for a minute or two, when she thought she slept, though a slight movement instantly aroused her; but, to use her own expression, she was "alive nowhere except the head and heart." Perhaps the best statement of the result from giving the bromide is afforded by still further quoting my observant and accomplished patient in an extract from a note from her: "I have been sending my thoughts back to the time when opium was my horror, and severe pain as easy to bear as its effects. If the pain was relieved, the faintness would return after twelve, fifteen, or twenty-four hours from the time of taking the opium. Now, on taking twenty grains of the bromide one-half hour before a dose of the watery extract, and again about two hours afterwards, I am pretty secure." From the first time in which, when giving her the bromide, its influence in preventing the unpleasant consequences of opium was noticed until the present bromide has not failed us once. On morphia it has least influence, and morphia and codeia always affected her the worst. Still it has an influence and four doses keep her tolerably comfortable.

A case as striking, though not one in which the observation has been as often repeated, is that of an old lady subject to the attacks of diarrhoea, and in whom all opiates, even paregoric, produced faintness, marked, though not so marked as in the preceding instance, but much more decided headache and nausea. By taking forty grains of bromide, in twenty grain doses, beginning about three hours before she takes opium, she bears perfectly well twenty-five drops of laudanum.

Of another case I transcribe the record, as kindly kept for me at the Hospital by my resident physician, Dr. James C. Wilson. It reads thus:

Annie C., Irish, aged 35, a domestic, widow. Admitted into the Pennsylvania Hospital Feb. 8th, 1871, suffering with anæmia and with impaired digestion, which, however, is not associated with any manifest organic disease; was placed upon tinct. ferri chloridi gtt. xx, t. d., and good diet.

Feb. 12th. Complains of sleeplessness; states that she was awake all last night. This report was corroborated by the night nurse. Was ordered liq. morphiæ sulph. f℥ij at bedtime.

13th. Passed a sleepless night. After taking the morphia she experienced a feeling of great weakness; felt dizzy and confused; described herself as "seeing all kinds of strange things." She had headaches as these phenomena passed away, with dryness of the throat and great restlessness, which lasted until morning. On rising she had intense nausea and vomiting, which continued until noon. 2 P. M., given potassii bromid. gr. xxx. 8 P. M., liq. morphiæ sulph. f℥ij as last night. Pulse 128, respiration 28, temperature 100°.

14th, A. M. Pulse 108, respiration 24, temperature $98\frac{3}{4}$°. States that she slept very well, and feels in every respect quite as well as usual. No dizziness nor headache; no nausea or vomiting followed the administration of the morphia.

17th, P. M. Pulse 116, respiration 20, temperature $99\frac{1}{2}$°.

18th, A. M. Pulse 96, respiration 29, temperature $98\frac{1}{4}$°. Had taken no medicine, except the tinct. of chloride of iron, since the evening of the 13th inst. She again complains of sleeping poorly. Last night she did not fall asleep until towards morning. 8 P. M. given tinct. opii deodorat. gtt. xxv. Pulse 83, respiration 20, temperature 99°.

19th, A. M. Pulse 76, respiration 20, temperature 99°. She states that after taking the medicine last night she felt weak and faint, was dizzy, and fancied that she saw curious and grotesque objects; had no pain in her head, but was restless, and had no sound sleep, although she dozed at times. Had a feeling of faintness and nausea on rising, but no vomiting. 6 P. M., took potassii bromid. gr. xxx; 9 P. M., tinct. opii deodorat. gtt. xxv.

20th. Fell asleep about midnight, and slept well till morning. Had some dizziness, but no feeling of faintness; no confusion or headache after taking the opiate. Had no nausea, vomiting, or faintness on rising.

I will briefly cite one more case, which was very recently under my observation.

A young lady, in whom opium produced a most decided faintness and nausea, was attacked with muscular rheumatism, and took laudanum at night to relieve her discomfort. She sent for me the next morning, and I found her with dry throat, giddy, and weak. Prescribing the bromides, partly to counteract the effect of the opium, partly for other reasons, she was enabled to take the opiate without the least inconvenience; and, when a few days afterwards I found that she had been resorting with impunity to an opiate of either Dover's powders or laudanum, every night, I ascertained that, by a misunderstanding of orders, she had continued to take the bromide mixture, in addition to the prescription of acetate of potassa and colchicum, which I had directed to replace it.

I might continue to multiply this narrative of cases, but it will not make the subject any clearer. I shall rather investigate the result, in some special directions, of giving the two remedies. The bromide does not destroy either the anodyne or the hypnotic effects of the opiate; on the contrary, it rather heightens both, and more particularly the latter. To quote again from the letter of my first patient—"The more bromide I take the sooner do I get sleep after a dose of opium. Two doses of bromide (twenty grains each) are not usually enough to counteract the exciting effects, and procure sleep under five or six hours from the time of taking." The faintness from opium is the phenomenon most markedly prevented; next in the readiness of being influenced stand the headache, vertigo, and nausea, then the itching of the surface, and dry mouth.

The bromide has seemed to me to act best when it is given some hours before the opium, and forty to sixty grains--generally forty grains—prove sufficient. But it also has an action, sometimes, however, markedly less, when combined with opium; and, should unpleasant consequences have accrued from this, the bromide will mitigate their severity. Even the cutaneous itching is favorably influenced, and I have known repeated doses most decidedly affect the faintness. When morphia is used hypodermically, it is then most necessary to give the bromide some time in advance, and it may take larger doses to accomplish the purpose. At least it has so seemed to me—though I have not often tested this, since most of the observations were made in persons who took opium by the mouth.

Now, though I think that the corrective influence of the bromide on opium holds good as a general truth, we meet at times with exceptions. Dr. Wallace, to whom I had mentioned the matter, told me that while he had in several instances obtained the most gratifying results, he had failed in one; and Dr. James C. Wilson has taken notes for me of a patient in the Pennsylvania Hospital suffering from advanced phthisis, in whom sleeplessness, a feeling of confusion, dizziness, and dull throbbing, frontal headache, and nausea and vomiting in the morning were caused by one drachm of the solution of sulphate of morphia. The addition of the same amount of spirits of chloroform obviated the unpleasant results, though it finally failed; and sixty grains of the bromide did not prevent one-fourth of a grain from producing the disagreeable consequences.

But these exceptions are not, I believe, numerous, and the bromide does not often disappoint. Of course, in investigating its value with reference to the questions here discussed, we have to test it on those with whom opium really disagrees, and not on such who merely say that it does; for, from some reason or the other, many persons seem to think it a point of honor to make this statement, though out of ten such persons nine are quite certain to be able to bear the anodyne as readily, and to derive as much advantage from it as the rest of mankind with whom it professedly does not disagree. Yet, considering the number of therapeutical applications of the invaluable drug, and the fact that we may be prevented from using it in instances in which its employment might be of the greatest moment, because to use it seems impossible, it will be of service to be able to control its action, and the remarks here made will, I trust, prove to represent the full truth. Moreover, they may be looked upon as a contribution towards a most interesting and comparatively neglected part of therapeutics, what may be called the *corrective influence* of one drug over the other; corrective in obviating its bad results, while not interfering with, but rather heightening the good ones.—*Am. Jour. Med. Sciences.*

